# Virtual Reality Hemifield Measurements for Corrective Surgery Eligibility in Ptosis Patients: A Pilot Clinical Trial

**DOI:** 10.1167/tvst.11.10.35

**Published:** 2022-10-25

**Authors:** Margarita Labkovich, Andrew J. Warburton, Stephanie Ying, Aly A. Valliani, Nicholas Kissel, Randal A. Serafini, Raj Mathew, Megan Paul, S. Malin Hovstadius, Vicente N. Navarro, Aashay Patel, Harsha Reddy, James G. Chelnis

**Affiliations:** 1 Department of Medical Education, Icahn School of Medicine at Mount Sinai, New York, NY, USA; 2 Department of Statistics & Data Science, Carnegie Mellon, Pittsburgh, PA, USA; 3 Nash Department of Neuroscience and Friedman Brain Institute, Icahn School of Medicine at Mount Sinai, New York, NY, USA; 4 Department of Medical Education, SUNY Downstate, Brooklyn, NY, USA; 5 Department of Uro Onc Research, Weill Cornell Medicine, New York, NY, USA; 6 Department of Ophthalmology, Icahn School of Medicine at Mount Sinai, New York, NY, USA

**Keywords:** ptosis, hemifield, perimetry, virtual reality, humphrey visual field analyzer

## Abstract

**Purpose:**

We developed an accelerated virtual reality (VR) suprathreshold hemifield perimetry algorithm, the median cut hemifield test (MCHT). This study examines the ability of the MCHT to determine ptosis severity and its reversibility with an artificial improvement by eyelid taping on an HTC Vive Pro Eye VR headset and the Humphrey visual field analyzer (HVFA) to assess the capabilities of emerging technologies in evaluating ptosis.

**Methods:**

In a single visit, the MCHT was administered along with the HVFA 30-2 on ptotic untaped and taped eyelids in a randomized order. The primary end points were a superior field visibility comparison with severity of VF loss and VF improvement after taping for MCHT and HVFA. Secondary end points included evaluating patients’ Likert-scaled survey responses on the comfort, speed, and overall experience with both testing modalities.

**Results:**

VR's MCHT superior field degrees visible correlated well for severe category margin to reflex distance (r = 0.78) compared with HVFA's (r = −0.21). The MCHT also demonstrated noninferiority (83.3% agreement; *P* = 1) against HVFA for detection of 30% or more superior visual field improvement after taping, warranting a corrective surgical intervention. In comparing hemi-VF in untaped eyes, both tests demonstrated relative obstruction to the field when comparing normal controls to severe ptosis (HVFA *P* < 0.05; MCHT *P* < 0.001), which proved sufficient to demonstrate percent improvement with taping. The secondary end point of patient satisfaction favored VR vision testing presentation mode in terms of comfort (*P* < 0.01), speed (*P* < 0.001), and overall experience (*P* < 0.01).

**Conclusions:**

This pilot trial supports the use of MCHT for the quantitative measurement of visual field loss owing to ptosis and the reversibility of ptosis that is tested when conducting a presurgical evaluation. We believe the adoption of MCHT testing in oculoplastic clinics could decrease patient burden and accelerate time to corrective treatment.

**Translational Relevance:**

In this study, we look at vision field outputs in patients with ptosis to evaluate its severity and improvement with eyelid taping on a low-profile VR-based technology and compare it with HVFA. Our results demonstrate that alternative, portable technologies such as VR can be used to grade the degree of ptosis and determine whether ptosis surgery could provide a significant superior visual field improvement of 30% or more, all while ensuring a more comfortable experience and faster testing time.

## Introduction

Blepharoptosis, or superior ptosis, is a drooping of the upper eyelid that causes an abnormally low-lying upper eyelid margin.^1^ Although the majority of cases are benign in etiology, ptosis can be the first indication of serious neurogenic conditions that require prompt medical attention. Ptosis can be congenital as a result of levator maldevelopment or acquired as a result of disease, levator muscle dysfunction, or facial trauma.^2^ Aponeurotic ptosis is the most common type of acquired ptosis and results from overstretching of the levator muscles, usually owing to aging.^3^

Ptosis is a problem for patients for aesthetic and functional reasons, because the condition creates a tired and aged appearance and can cause visual field obstruction. It is most often diagnosed with standard eyelid measurements and visual field analysis.^4^ Anatomic drooping grade is assigned based on margin to reflex distance (MRD1), or the distance between upper lid margin and reflex. It is graded as follows: mild (3–4 mm), moderate (2–3 mm), or severe (<2 mm), with ptosis resulting in superior visual field (SVF) loss ranging from 0% to 80% (0°–40°).^5^^,^^6^ Corrective surgeries, such as blepharoplasty, have been functionally beneficial to patients with an SVF loss of 12° or greater, which is evaluated using Humphrey visual field analyzer (HVFA) perimetry testing and is a key objective metric in determining whether a person qualifies for surgery.^7^

To quantify SVF loss, oculoplastic surgeons use a variety of methods in their practice or through referrals to general ophthalmologists, including MRD1 measurements, tangent screen testing, Goldmann kinetic perimetry, and Humphrey perimetry.^8^^,^^9^ Although Goldmann and Humphrey perimetry are the gold standard diagnostic instruments for quantifying SVF loss, the equipment is costly and requires a trained technician in addition to a designated dark room space in the medical office to run the tests. They are also time intensive, because testing both eyes (without and with tape) with these machines takes 10 ± 2 minutes and 50 ± 10 minutes, respectively.^10^ Additionally, patients do not find the experience pleasant, often reporting significant discomfort and confusion without intensive coaching, which impacts their results. A study showed that repeated perimetry testing improves 30-2 HVFA performance in patients with newly diagnosed glaucoma owing to a learning curve that leads to unreliable results.^11^ Alternatively, some physicians opt for tangent screen testing, which entails a screen mounted on the wall for target detection in the central field. The test is faster, but does not generate an automated electronic output and, therefore, is not a consistent or sufficient tool for recording and reporting the degree of ptosis severity and any improvements.^8^ These limitations demonstrate the need for an accurate, portable, affordable, rapid, and user-friendly perimetry device for deployment within oculoplastic practices.

Virtual reality (VR) devices have recently gained attention in the vision care field as cost-effective screening tools.^12^^,^^13^ VR-mediated perimetry is a promising strategy for visual field testing in ptosis patients, thereby providing an alternative device solution that is potable and has a lower footprint in identifying patients who would benefit from undergoing corrective surgery, while also allowing for automated record keeping and focusing on patient comfort. Furthermore, HTC Vive Pro Eye integrated eye tracking allows for measurement of gaze deviations and invalidation of eye motion-sensitive tests that are incorrectly attempted.^14^ Several studies have demonstrated noninferiority of VR-mediated perimetry to equivalent Humphrey perimetry examinations.^14^^–^^16^ However, to our knowledge, no group has developed and validated an accelerated suprathreshold hemi-VF perimetry MCHT test that relies on meridian mapping in patients with ptosis.

## Methods

### Clinical Trial Design and Workflow

Study participants were recruited from oculoplastic clinics at New York Eye and Ear Infirmary of Mount Sinai from November 2019 through December 2020. Patients were eligible if they were at least 18 years of age and were able to understand and provide verbal and written informed consent. The experimental group included patients with physician-identified ptosis and control group patients were not eligible if they had ocular surgery less than six months ago or contraindications for VR visual field testing, including anxiety disorder, seizure disorder, vertigo, or balance issues. This study was reviewed and approved by the Institutional Review Board of Mount Sinai (IRB #19-00670). Informed consent was obtained from participants after explanation of the nature, details, and risks of the study in their plain, native language. All participants were informed they could opt-out at any time without recourse.

**Figure 1.**
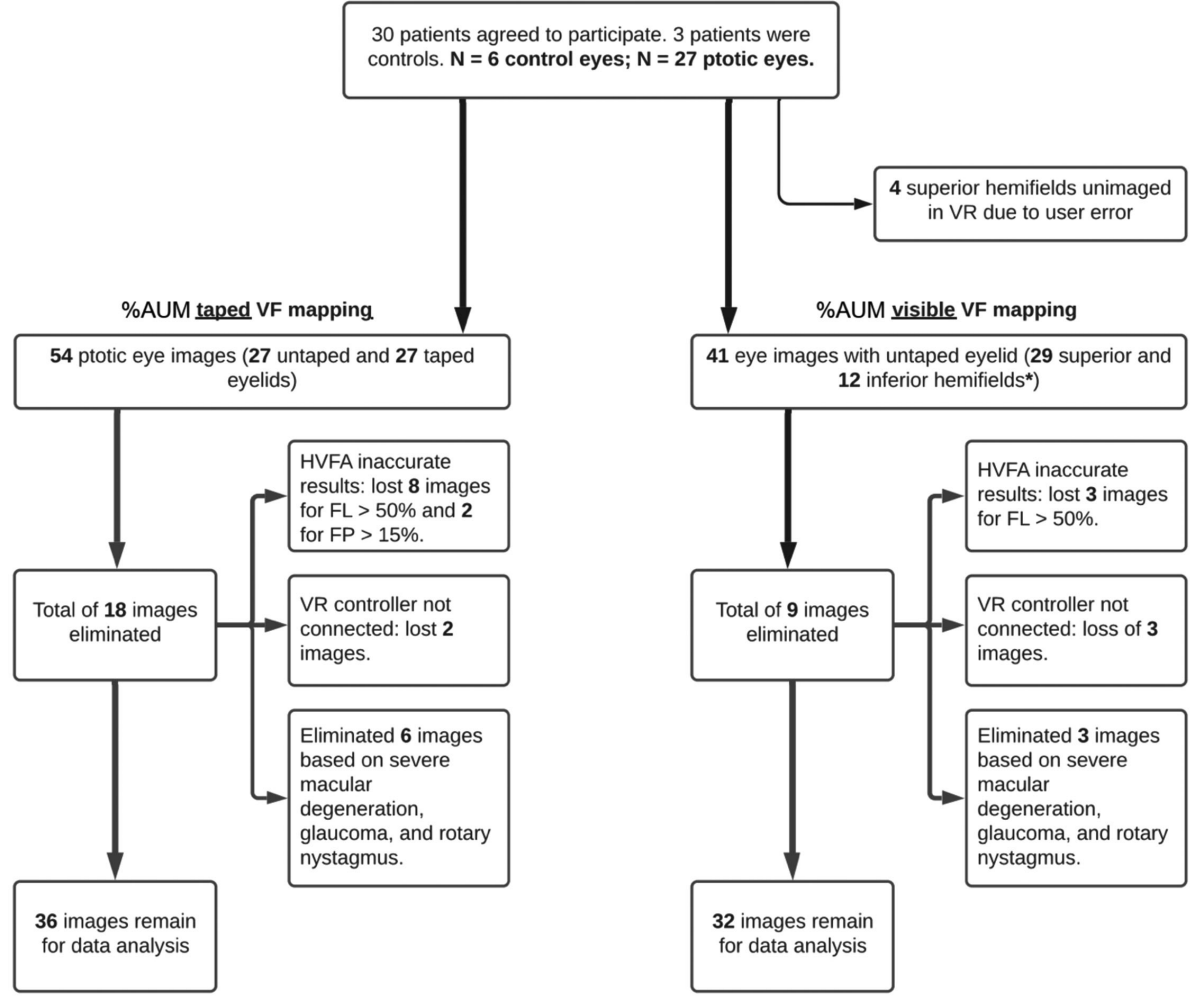
Flow diagram of data selection process for uncorrected and corrected visual field testing in ptosis patient population. FL of 50 or more, false-positive (FP) rate of 15 or greater. *Inferior fields were obtained in patients with eyelid droop below the horizontal meridian.

The sample size for the study is 20 eyes, which is in line with previous research on comparing MCHT perimetry outputs to Humphrey MATRIX visual field and Swedish interactive threshold algorithm in glaucoma patients and healthy controls.^17^^,^^18^ Assigned MRD1s were evaluated by at least two oculoplastic ophthalmologists. The clinical recruitment and visual field mapping pipeline are outlined in Figure 1.

The most common reasons for opting out were a) being unable to stay after their scheduled visit or b) not being interested. Demographic information of each patient who successfully completed the study can be reviewed in Table 1.

**Table.** Demographic of Study Participants Whose Data Were Included in the Final Analysis

Demographics	No. (%)
Gender	
Female	17 (85)
Male	3 (15)
Age, years	
<30	1 (5)
30–60	7 (35)
>60	12 (60)
Vision refraction	
Yes	8 (40)
No	12 (60)
Ocular conditions	
Cataract	2 (10)
Glaucoma	2 (10)
Macular degeneration	1 (5)
Rotary nystagmus	1 (5)
Diabetic retinopathy	None (0)
Chronic diseases	
Hypertension	3 (15)
Diabetes	3 (15)
Inflammatory vasculitis	1 (5)

**Figure 2.**
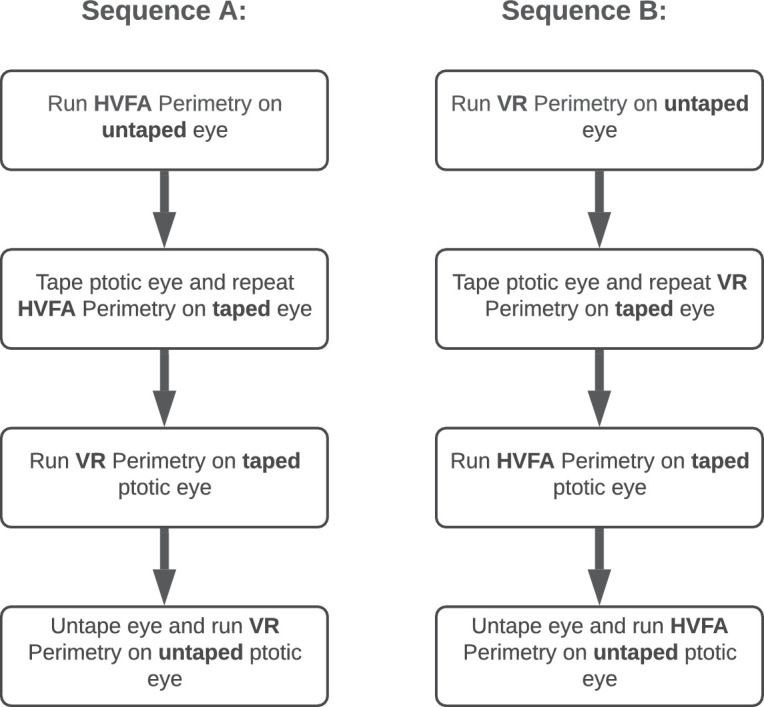
Flow diagram depicting randomly assigned sequences A and B for the minimalization of technology-specific confounding variables.

Visual fields of untaped ptotic eyes represent the patient's visual field at baseline with each patient serving as their own control. Each ptotic eye was also taped, which represents the patient's corrected visual field. Patients were randomized to one of two test sequences of receiving the MCHT then HVFA or vice versa to control for fatigue and recency bias as confounding variables. Sequence A consisted of an untaped perimetry exam using HFVA, taping the eye and repeating the exam on the HFVA, leaving the eye taped for MCHT testing, and untaping the eye and repeating the MCHT exam. Sequence B was the reverse order of Sequence A: untaped MCHT exam, taped MCHT exam, taped HVFA exam, and untaped HVFA exam. Each sequence order is depicted in Figure 2 with equal balancing of participants. HVFA testing was done on the research floor of NYEE by a trained clinical researcher along with MCHT perimetry that was also stationed on the same floor.

After undergoing the MCHT and HVFA perimetry testing, all participants completed a Likert-scaled survey evaluating speed, comfort, and overall experience with both technologies. They also provided information regarding the frequency and convenience of their visits to an ophthalmology practice. From 27 people who agreed to participate, 23 participants completed the survey.

**Figure 3.**
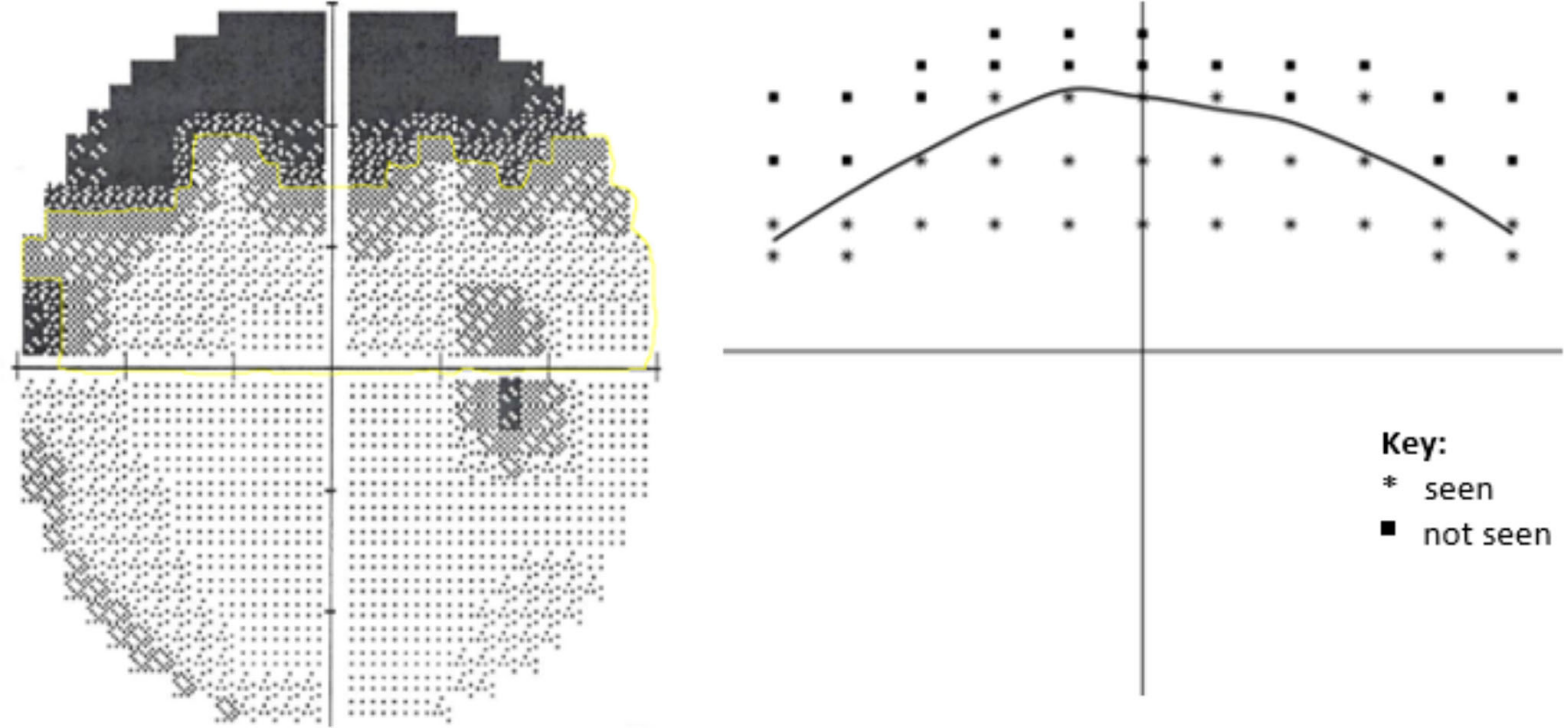
(*Left*) HVFA 30-2 visual field output with a yellow meridian tracing of visible SVF. (*Right*) SVF algorithm VR output after median cut algorithm processing to approximate the AUM.

After a participant's HVFA visual field data was collected it was deidentified and assigned a randomly generated study ID number. To measure area under the meridian (AUM), each field was traced using ImageJ software to calculate the visible area of the visual field and percent change between untaped and taped eyes. Three independent raters blinded to ptosis severity rating were used to manually trace out the area using ImageJ and blinded to each other's results, which were averaged to give a final value used in statistical analysis. The grayscale cutoff threshold used for this study was <10 Apostilibs. MCHT visual field data stored under a study ID was automatically converted to produce an output similar to that of HVFA shown in Figure 3.

The area of a participant's MCHT-generated hemi-VF was quantified using an R script (https://github.com/RetinaTechnologies/data-visualization). The accuracy of this code's superior meridian tracing was evaluated in patients with ptosis using manual tracings with ImageJ software (r = 0.99), which can be seen in Supplemental Figure S1.

### Median Cut Hemifield Test

The median cut hemifield test (MCHT) is a recursive algorithm that incrementally maps out a meridian in the superior aspect of the visual field. The SVF is divided through Cartesian space into rows and columns—each row 12° separated from each other. The algorithm places the start point in the middle of a randomly selected column. If a user confirms that he or she has seen it with a trigger response, the algorithm places a new point half-way between the highest row and the last-tested point. If the point was not recorded, the algorithm places the subsequent point half-way between the former point and the lowest row. The stop condition is triggered when the old and new pivots differ by 10 or fewer pixels. Each column tests an additional point above and below the stop condition coordinates to ensure fair mapping of the meridian to avoid incorrect deviations (i.e., scotomas). This process is repeated for every column along the meridian and is run twice, with and without taping the user's eyelids. The user's SVF is calculated as the area under the meridian via the trapezoidal rule and a percent difference is calculated to quantify the level of ptosis. Overall, the entire algorithm runs in logarithmic time.

### Methods for Degrees Visible

**Figure 4.**
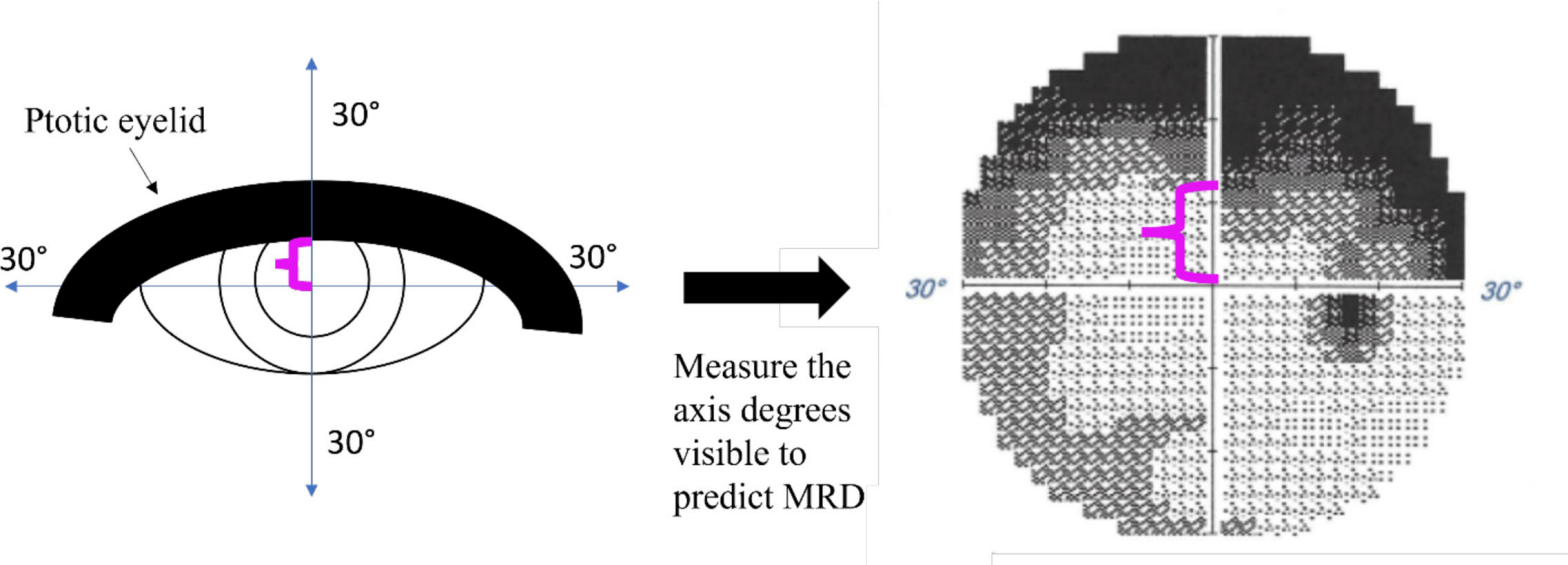
Schematic of the determination of *y*-axis degrees and prediction method for MRD1 measurements in patients with ptosis. The measurement of the meridian at less than 10 Apostilibs on the Humphrey grayscale map was predicted to correlate with the MRD1 measurements.

Given the current literature, MRD and percent visible denoted by the AUM of the SVF have had mixed levels of association; thus, a novel methodology of predicting MRD1 was conducted by analyzing the percent degrees visible along the *y*-axis (*x* = 0) as an equivocation for the relative degrees obscured by the eyelid.^19^^–^^21^ The percent degrees visible was calculated by taking the degrees visible (>10 Apostilibs on HVFA grayscale) at *x* = 0 (if the left and right side had different degrees visible, then the average was obtained) as described in Figure 4. Of the 20 eyes, there was 1 outlier that exceeded the definition of an outlier as it was more than 1.5 times the Interquartile range and was thus removed from the analysis.

### Data Analysis

There were two coprimary end points in this study. The first was agreement between the area of the untaped SVF and ptosis severity grading as measured by MCHT and the HVFA, which was assessed using percent superior hemifield visible and perimetry degrees visible. The second was agreement between the percent vision improvement after taping the eyelid as measured by MCHT and HVFA. Data analysis was performed by an independent statistician and all statistics were performed in R version 4.0.4. To evaluate the first coprimary end point, the median percentage of the visual field that was visible in the untaped eye was compared between the MCHT and HVFA using the Wilcoxon signed rank test.

For the primary end point on determining the MCHT suprathreshold perimetry ability to determine the degree of severity, the superior hemifield was measured using both visible visual field (measured by AUM) and percent degrees visible. A correlational analysis was done between the two to demonstrate the ability of percent degrees visible to serve a proxy to conduct MRD1 grading comparisons. This method of assessing superior hemifield function accounts for the field irregularities in meridian tracing. Afterward, percent degrees visible was correlated with mild and severe ptosis cases using fractional analysis—splitting the patients into severe (MRD1 of <2 mm) and mild (MRD1 of ≥2 mm) ptosis and treating each group as an independent cohort. These cohorts were reported with independent correlations and *P* values for both MCHT and HVFA to compare the percent degrees visible versus the MRD1 scores. Normal eyelid MRD1 scores (MRD1 = 5) were not included in the fractional analysis because the MCHT and HVFA both returned 0% change in visual field and 0% change in percent degrees seen; thus, correlations could not be drawn.

The percentage of the visible visual field was then correlated with the degree of severity of the ptosis (categorical) and MRD1 measurements (continuous). For severity grading, normal was defined as an MRD1 of 4 mm or more, mild 2 to 4 mm, and severe less than 2 mm. The Kruskal–Wallis rank-sum test, also known as a nonparametric analysis of variance, was used without assuming a continuous normalized t-distribution to compare the distribution of the percentage of visible visual field among severity grades for MCHT and HVFA. This test was used to determine whether the medians of different groups were the same or not.

In a post hoc analysis of VR results, Conover's test was used to evaluate pairwise differences between each ptosis severity category with Holm's adjustment method for *P* values.^19^ To evaluate the second coprimary end point, agreement between the MCHT and HVFA test based on percent vision improvement after taping, a 30% improvement threshold after taping was set. This threshold represents the improvement required between the untaped and taped visible visual fields to qualify for ptosis corrective surgery. Fisher's exact test was performed to identify whether the test outcomes are independent.

The accuracy, true positive rate, and precision (or percentage of predicted positives that are correct) of the tests were evaluated to determine bias toward one type of testing modality (MCHT or HVFA) in disagreeing data points using McNemar's test. To test our accuracy against a 90% accuracy or larger set as a null hypothesis, an exact hypothesis test was performed, which is a one-sided binomial test. We calculated the power and sample size based on number of ptosis eyes that qualified for this end point analysis as outlined in Figure 1.

## Results

### Untaped Visual Field Comparison

The first primary end point represented the untaped percent visible AUM data analysis grouped by using both MRD values and ptosis severity categories, using the following definition of normal as an MRD1 of 4 mm or more, mild 2 to 4 mm, and severe less than 2 mm. Data were compared between each respective group using MCHT and HVFA perimetry studies. The second primary end point represented percent difference AUM with taping of the eyelid.

In analyzing the first primary endpoint, majority of patients recruited were suffering from severe ptosis (MRD1 of <2 mm). A total of 30 eyes were included with 18 (62.5%) being severe ptosis, 6 eyes (18.75%) classified as mild, and 6 eyes (18.75%) with normal MRD1 values.

**Figure 5.**
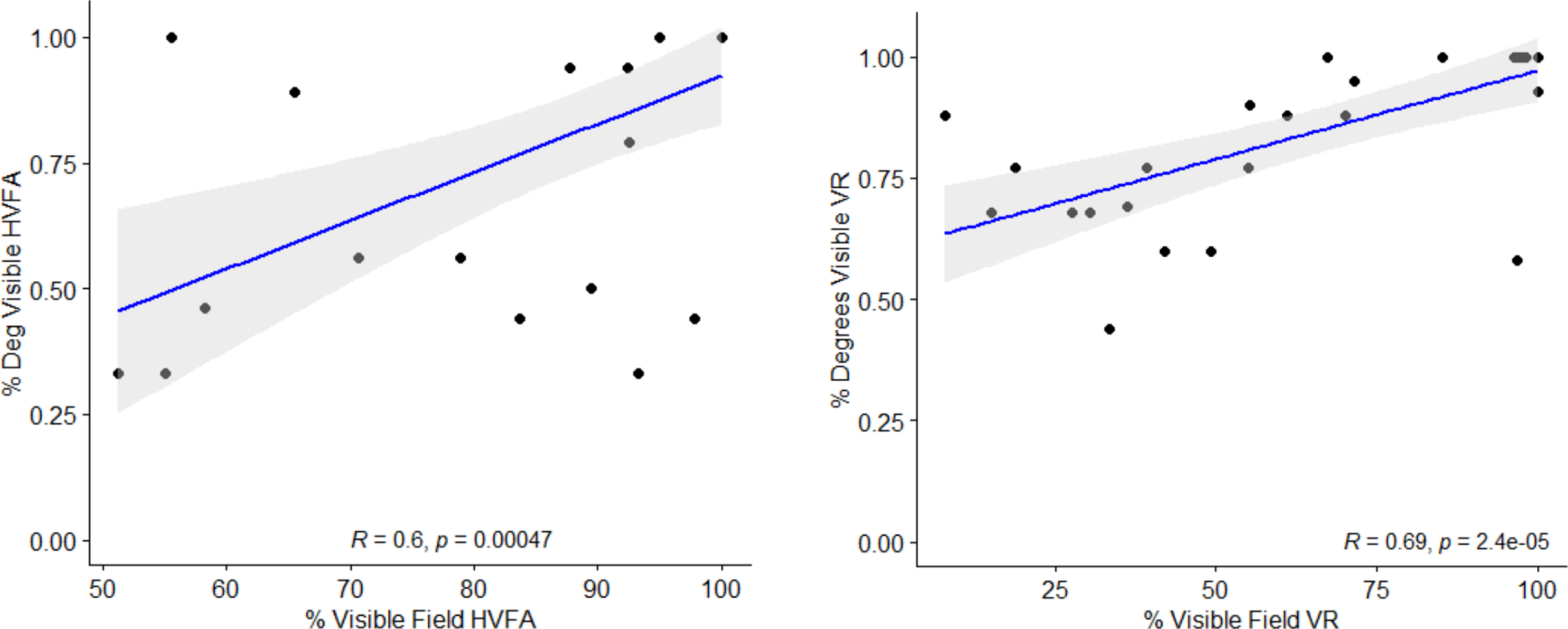
Correlations between percent visible field and percent degrees visible for VR and HVFA. The percent degree visible was determined by dividing the degrees seen at *x* = 0 by the maximum possible visible degrees seen in the test (*N* = 30). Given the overlap in values, the number of points varies between the two graphs with 14 points overlapping on the left graph and 8 points on the right graph. Consistent statistically significant positive correlations between the percent degrees visible and percent visible field provides confidence in the association between the two independent measurements.

Intrapatient HVFA and MCHT hemi-VF up-taped readouts percent visible superior field was correlated with percent degrees visible (Fig. 5) with HVFA (r = 0.6; *P* < 0.001) and MCHT (r = 0.69; *P* < 0.001). Thus, the measurements for percent degree and percent visible were statistically significant across ptosis severity gradients and moderately positive in both HVFA and MCHT perimetry testing.

**Figure 6.**
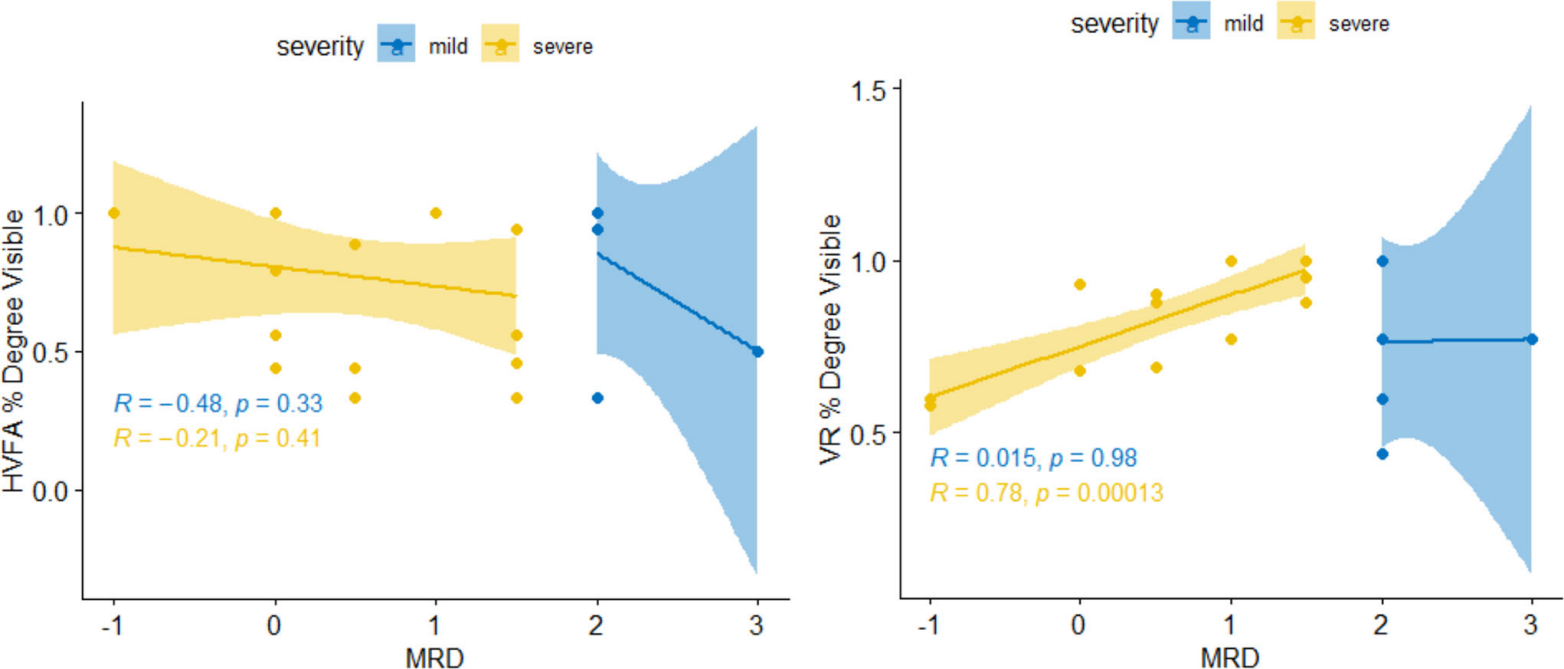
Fractional analysis of HVFA and VR for mild to severe ptosis (MRD1 of ≤2) with a sample size of 24. Both the right and left graphs have a 7-point overlap. The left demonstrates HVFA percent degrees visible versus MRD1 scores divided by severe (MRD1 of <2 mm) versus mild ptosis (MRD1 of ≥2 mm). The HVFA had weak negative correlations for both the severe and mild ptosis groups (r = −0.21 and −0.48, respectively, both of which were not significant). The right demonstrates VR percent degrees visible versus MRD1 scores divided by severe (MRD1 of <2 mm) versus mild ptosis (MRD1 of ≥2 mm). The VR had significantly strong positive correlations between percent degrees visible and MRD1 for severe ptosis patients (r = 0.78; *P* = 0.00013), but poor positive, not statistically significant correlations for mild ptosis (r = 0.015; *P* = 0.98), demonstrating that VR has stronger associations between percent degrees visible versus MRD1 scores.

Subsequently, the percent degree visible was compared with MRD1 in the mild to severe range using fractional analysis (Fig. 6), which showed HVFA (r = −0.21; *P* = 0.41) for severe MRD1 values and MCHT (r = 0.78; *P* < 0.001). Mild severity did not correlate well for both technologies with HVFA (r = −0.48; *P* = 0.33) and MCHT (r^2^ = 0.015; *P* = 0.98).

**Figure 7.**
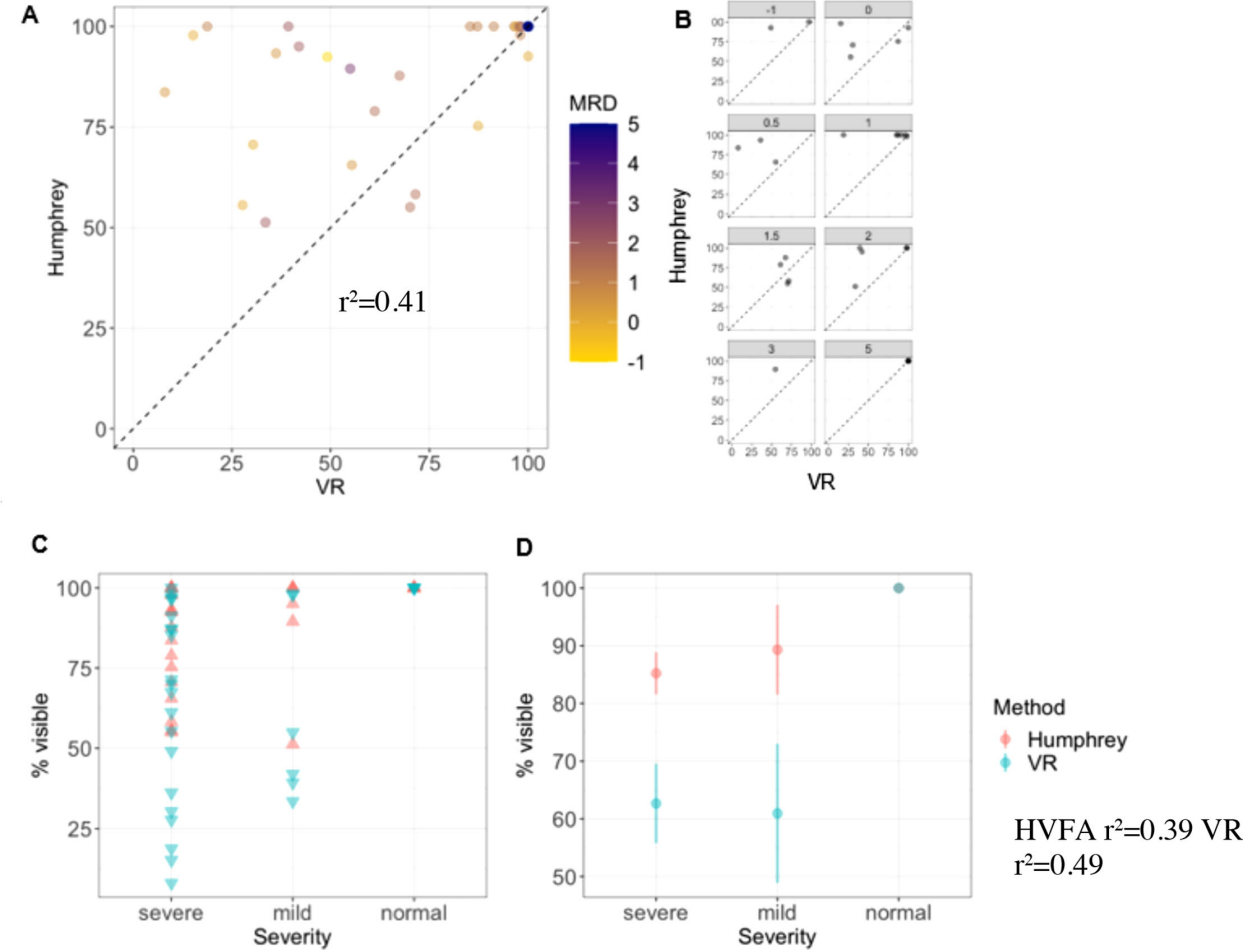
(A) Percent visible field AUMs mapped as scatter plots of HVFA and VR tests with an MRD heat map. (B) *Dotted diagonal lines* with data points on the line indicating a perfect agreement between two tests, points above the dotted line have lower VR than HVFA percent visible AUM and points below the *dotted line* have higher VR percent visible AUM than HVFA. Each data point represents a percent visible AUM value from one eye. (C) Scatter plot of each AUM classified by severity. (D) Averaged percent visible AUM values for HVFA and VR tests.

An additional analysis of the superior hemifield data is shown in Figure 7, with datapoints left of the axis representing a higher percent visible VF rating on the HVFA than SVF. Overall, visual field measurements were underestimated by MCHT when compared with HVFA. The distribution of these points by MRD1 demonstrates that MCHT underestimations were independent of ptosis severity. Coefficient of determination between visibility percentage and MRD for both tests are r^2^ = 0.39 for HVFA and r^2^ = 0.49 for MCHT (Fig. 7). The coefficient of determination between MCHT and HVFA is r^2^ = 0.41 (Fig. 7). A Wilcoxon signed rank test demonstrated statistical significance between the two device groups (*P* < 0.001).

When looking at the difference in the mean AUM between severity groups, HVFA untaped visual field readouts were significant in differentiating between severe, mild, and normal ptosis gradings (Kruskal–Wallis rank-sum test: χ^2^ = 7.35; *P* < 0.05). The same comparison with MCHT visual field readouts also yielded significantly different mean AUM values among ptosis severity groups (Kruskal–Wallis rank-sum test: χ^2^ = 13.25; *P* < 0.01). Across the severity levels, HVFA's outputs showed significant difference between normal and severe groups (Conover's test: *P* = 0.016), whereas normal to mild group comparison (Conover's test: *P* = 0.19) and mild to severe (Conover's test: *P* = 0.4) were not significant. For MCHT test outputs, a significant difference was found between normal eye visual field AUMs and those of both mildly (Conover's test: *P* < 0.01) and severely (Conover's test: *P* < 0.001) ptotic eyes with the MCHT approach. However, as with HVFA, the MCHT readouts were not significantly different between mild and severe groups (Conover's test: *P* = 0.98). It is important to note that, although the MCHT hemi-VF approach yielded higher sensitivity for detecting ptosis and differentiating MRD1 of severe category based on percent degree visible, a larger trial is necessary to better power these measurements.

### Taped Visual Field Comparison

A total of 36 images from 18 eyes, once untaped and once taped, were selected for this end point after quality control exclusions. Of these eyes, 5 (27.8%) were graded as mild, and 13 (72.2%) were graded as severe.

**Figure 8.**
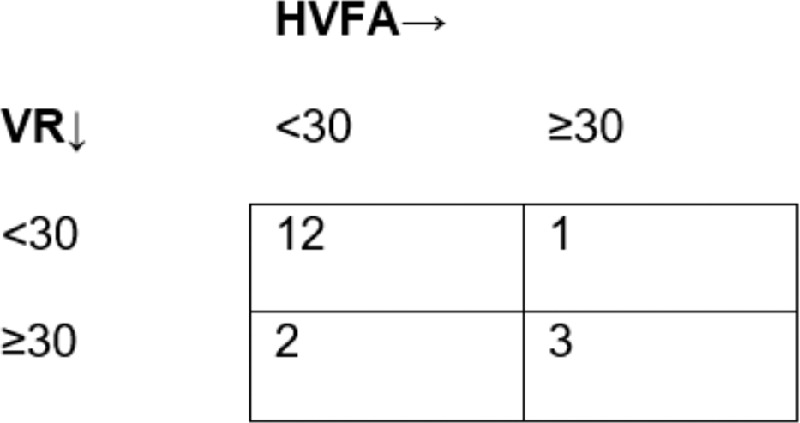
Matrix demonstrating agreement between VR and HVFA in a 30% or greater AUM increase with taping of the eyelid. The matrix cells contain the number of eyes that fell into each respective category.

The second primary end point focused on comparing the ability of these two technologies to detect a 30% or greater simulated correction of ptotic visual field losses by performing eye taping—as it is done in a presurgical evaluation. As seen in the matrix plot in Figure 8, agreement between the MCHT and HVFA tests was 83.3%. The data points of disagreement are not significantly biased toward one technology (McNemar Test; *P* = 1). True positive rate for SVF was 75% with a precision of 60%. The SVF and HVFA were not significantly different at identifying a 30% or greater correction in visual field (exact hypothesis test: H_o_ ≥ 0.9; true accuracy, *P* = 0.27). The SVF and HVFA visual field correction outputs demonstrated a significant association (Fisher's exact test: *P* < 0.05).

### Participant Feedback Survey

**Figure 9.**
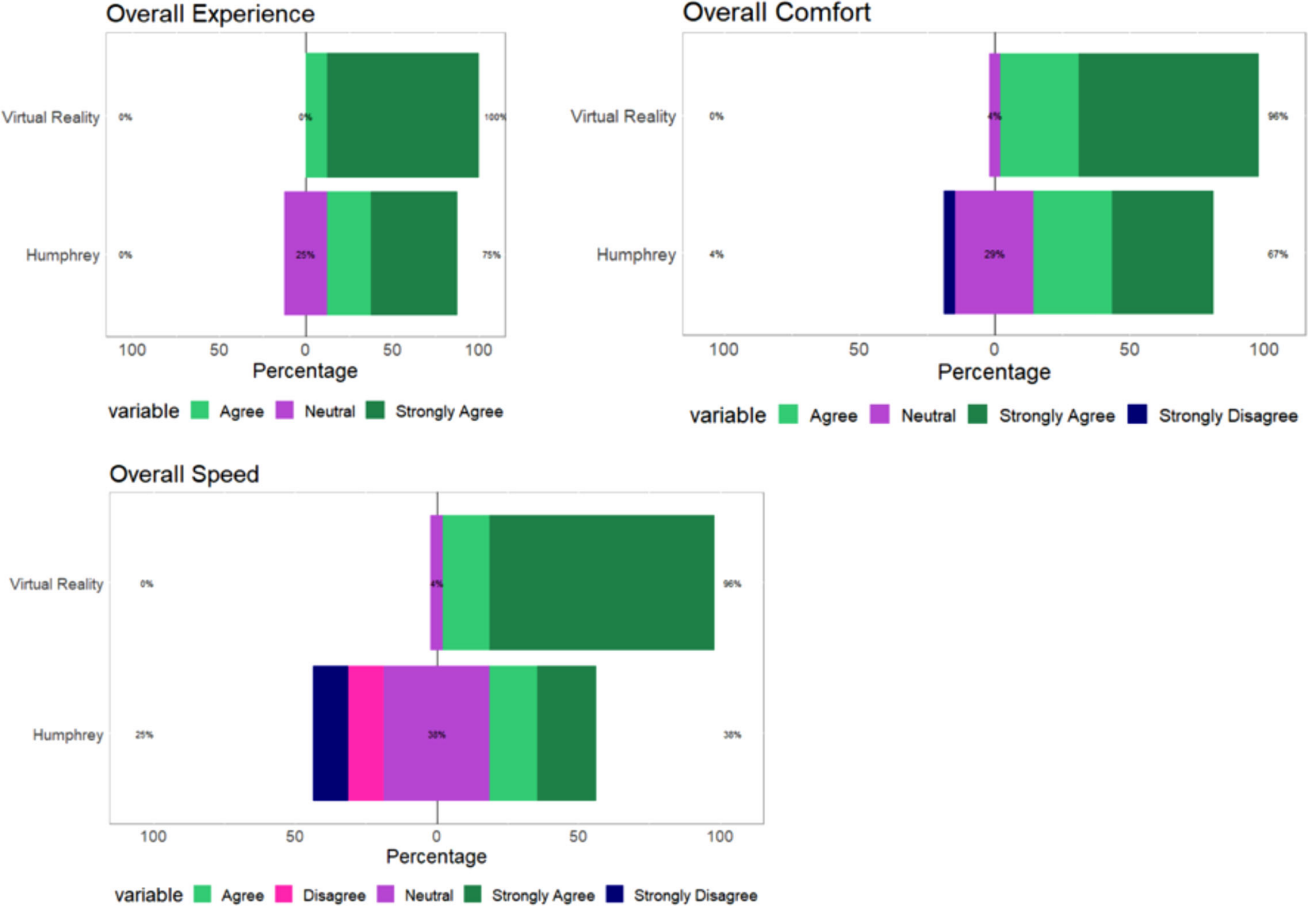
Likert graphs demonstrating patients’ ratings of HVFA and VR parameters based on survey data obtained after participants finished both HVFA and VR examinations focusing on three principal characteristics of the tests: speed, comfort, and overall experience. The VR examinations consistently scored higher (higher = better) compared with the HFVA for all three characteristics.

A total of 23 patients recruited for the study participated in the survey (76.7% participation rate), evaluating their experiences based on speed, comfort, and overall experience. The results are displayed as Likert graphs in Figure 9. The most common reason for not participating was having to leave the office.

**Figure 10.**
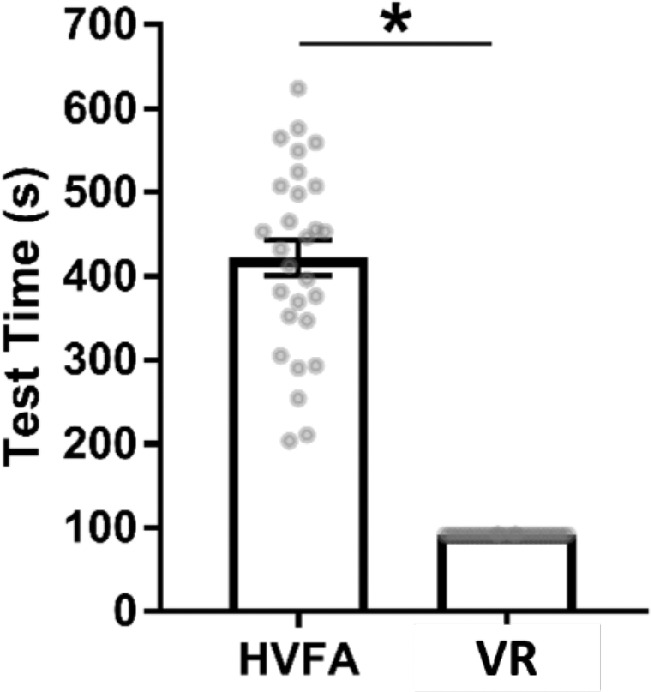
Average test times for the HVFA versus MCHT (VR) demonstrates the VR tests are significantly shorter than the Humphrey, averaging 91.2 seconds compared with the Humphrey averaging 421.5 seconds per eye.

According to the survey data, 96% of participants rated VR presentation mode as 4 out of 5 and 5 out of 5 or agree and strongly agree for its speed and comfort and 100% for experience. HVFA received 38% for speed and 67% for speed, with 75% for overall experience. Each device automatically recorded the time taken per test, demonstrating MCHT algorithm being consistently faster than the HVFA, averaging 91.2 seconds per eye compared with 421.5 seconds respectively (unpaired *t*-test, *P* < 0.001) (Fig. 10). When accounting for the fact that the HVFA is a full-field perimetry test, its half-field speed would be 210.7 seconds, which is still significant (unpaired *t*-test, *P* < 0.001) compared with the MCHT's superior field. A statistical of analysis of these results showed that all three parameters were significant in favoring VR experiences regarding comfort (Wilcoxon, *P* < 0.01), speed (Wilcoxon, *P* < 0.001), and experience (Wilcoxon, *P* < 0.01).

## Discussion

Given the continued need to quantify SVF correction in patients with ptosis and the lack of low-profile alternatives to HVFA, this study considered using VR-based MCHT perimetry testing to map out the SVF and its correction. In this pilot study, our group found that MCHT can serve as an alternative ptotic SVF-measuring technology for preoperative severity assessment.

Considering that MRD gradings are subjective and have a high degree of intra- and interobserver variability, we decided to additionally compare percent visible AUM to percent degrees that are visible (or light scale) within the total 30-2 perimetry field.^22^ This measurement also aims to bypass the difference in scale between the outputs from MCHT and HVFA examinations.^23^ Furthermore, this type of analysis can provide future insights into equivocating visual field outputs to predict MRD1.

Comparing untaped visual field AUMs, we saw that both HVFA and MCHT have a moderate positive correlation between percent visible field AUM and percent degrees visible, which indicated that the latter can be used as a proxy to objectively measure superior vision function detected by each device (Fig. 5). Both the HVFA and the MCHT had no deviation when reporting normal patients (MRD1 = 5mm) as they both reported 0% change in the percent visible field and the percent degree visible analysis (Fig. 6).

The results of this pilot trial suggested that MCHT has a higher correlation and potential sensitivity to lower MRD1 scores for severe ptosis compared with the HVFA analysis (Fig. 6). Additionally, the observed trend of MCHT outputs having a comparatively higher correlation can be also noted in Figure 7, where both testing modalities demonstrate statistically significant differences in comparing severe ptosis to normal controls. Notably, MCHT's outputs had lower AUMs in the mild category than HVFA's. MCHT consistently produced lower AUM values across all ptosis grades compared with HVFA.

One possible explanation for the MCHT's AUM values being lower is the ability of HVFA's forehead bar to artificially elevate upper eyelid muscles when the head is resting, causing an artificial improvement of ptosis. VR headsets, in contrast, are periorbital and do not support the eyelid with a forehead bar. However, given that the overall percent visible and MCHT to HVFA correlations are low, it remains possible that the inherent design of the tests, from a software perspective, could differ in a way that leads to inconsistent superior field values between the technologies, making it challenging for the two tests to agree on MRD grades.

A larger follow-on trial will be necessary to determine whether this study's findings are replicable in a larger population, and whether there is a clinical significance of differences between the MCHT and HVFA. Of note (discussed elsewhere in this article), the two technologies still produced similar rates of improvement after taping, suggesting a good delta sensitivity for both the HVFA and MCHT.

When considering the most deterministic criterion for ptosis corrective surgery, the 30% or greater correction reimbursement threshold, we found noninferior performance with high accuracy in determining percent improvement between the HVFA-measured change in the AUM after taping of the eyelid and that of the MCHT. Although only four taped patients with severe ptosis (31%) were eligible for corrective surgery with HVFA measurement (as determined by having a baseline visual field of <70% intact), seven patients who would qualify for surgery owing to severe ptosis were eliminated owing to HVFA's outputs falling outside of the inclusion criteria (Fig. 8). Of note, most patient exclusions from this study resulted from fixation loss (FL) of 50 or more and a false-positive rate 15 or more of HVFA's outputs. According to Zeiss, the recommended cut-off for the false-positive rate is 15% or more and an FL of 20% or more. Regarding FL, given substantial discomfort of undergoing a series of perimetry tests with both ptosis obstruction and taping of the eyelids, criteria of under 50% FL was considered satisfactory, and is further corroborated by other studies that exceeded 20% FL criteria.^10^^,^^24^

The accuracy of vision tests is highly reliant on patient performance, which is influenced by factors such as the simplicity of the tests and comfort. Using a Likert-scaled survey, we found that patients showed a significant preference for VR in terms of speed, comfort, and overall experience when comparing percentages of agree and strongly agree between technologies (Fig. 9). Overall, the results from both taped and untaped analyses demonstrate accuracy in terms of measuring relative vision obstruction based on the degree of severity, allowing physicians to quantitatively assess ptotic eyes for corrective surgery.

The MCHT algorithm was consistently faster than the HVFA, as demonstrated in Figure 10, which was true for before and after adjusting for HVFA being a full-field test. With similar results for mild ptosis, but improved correlations for percent degrees visible versus MRD1, VR is a viable alternative to HVFA for rapid and accurate screening for patients with ptosis and no other ocular conditions. Given the outcomes of this pilot study, future trials need to recruit a larger number of patients, especially in the mild ptosis range and those who present with ptosis in the setting of advanced ocular disease that may reduce their peripheral vision (such as glaucoma, diabetic retinopathy, etc.).

Along with an improvement in patient experience, introducing a rapid and portable SVF perimeter in oculoplastic practices allows for an alternative method of surgery eligibility determination that is more affordable and accessible, thereby increasing the chances for improved patient access. Based on this study, VR-based MCHT may serve as a viable alternative method for ptotic SVF measurements that meets preoperative screening requirements.

## Conclusions

Patients with ptosis undergo perimetry VF testing as an objective measure that shows whether their functionality is impaired enough to yield 30% or greater improvement after artificial correction by taping of the upper eyelid. Although HVFA is a currently accepted gold standard to perform VF perimetry, new technologies such as VR provide an alternative portable solution for MCHT hemi-VF testing in patients with severe ptosis. In this study, MCHT perimetry testing performed similarly to HVFA in demonstrating whether SVF improvement was sufficient enough to qualify the patient for corrective surgery. Before taping, the MCHT was successful in assigning MRD1 for severe ptosis cases. Data analysis from the feedback survey demonstrated a strong preference for the MCHT test in terms of speed, comfort, and overall experience, and the timing reports further demonstrated VR's time efficiency, proving its useful application in patients with ptosis who require a surgical intervention, particularly in specialized settings such as oculoplastic clinics.

## Supplementary Material

Supplement 1
