# Supplementary material for: Virtual Reality Hemifield Measurements for Corrective Surgery Eligibility in Ptosis Patients: A Pilot Clinical Trial
Source: Transl Vis Sci Technol. 2022 Oct 25;11(10):35. doi: 10.1167/tvst.11.10.35 (PMC9617504; doi:10.1167/tvst.11.10.35)

Supplemental Figures

Figure 1: Superior meridian tracing R script validation to ensure the R script accuracy as an AUM measurement tool. Its percent AUM was compared to the manual ImageJ tracings. Correlation between the two outputs r=0.99.

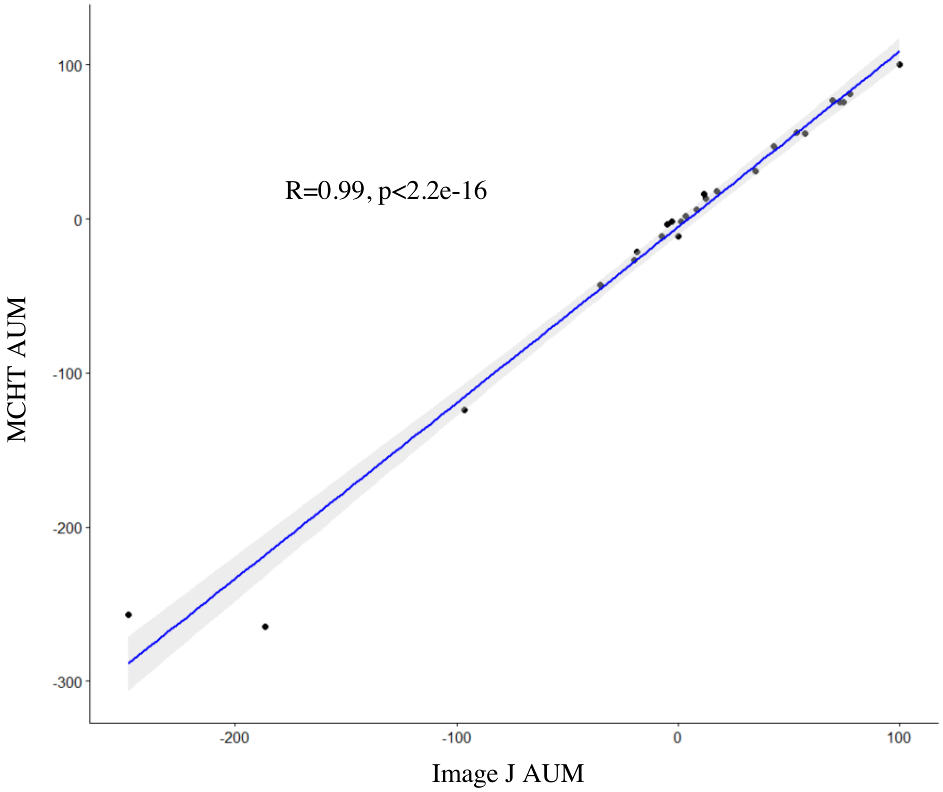

Supplement: Supplement 1 [file tvst-11-10-35_s001.pdf]
